# An unusual case of polyp with multiple appears honeycomb‐like depressions

**DOI:** 10.1002/deo2.70106

**Published:** 2025-04-01

**Authors:** Makoto Koda, Ikuro Koba, Taku Morita, Kazuhide Shimamatsu, Araki Toshihiro, Takumi Kawaguchi, Takuji Torimura

**Affiliations:** ^1^ Department of Gastroenterology Omuta City Hospital Fukuoka Japan; ^2^ Department of Gastroenterology Yamaga Central Hospital Kumamoto Japan; ^3^ Department of Diagnostic Pathology Omuta City Hospital Fukuoka Japan; ^4^ Division of Gastroenterology Kurume University School of Medicine Fukuoka Japan

**Keywords:** colon polyp, crypt, goblet cell, inverted sessile serrated lesions, magnifying endoscopy

## Abstract

The patient is a 63‐year‐old woman. She underwent lower gastrointestinal endoscopy at her local doctor, and a serrated lesion with a honeycomb‐like depression in the surface mucosa of the ascending colon measuring 35 mm in size was observed. The lesion was thought to be a sessile serrated lesion (SSL), but since there was a lot of mucus and it was difficult to observe the inside of the depression, SSL with dysplasia was also mentioned as a possible diagnosis. The patient was referred to our department and underwent endoscopic submucosal dissection of the colon. The pathological results showed no dysplasia component, and the diagnosis of SSL with inverted growth was made. Inverted SSL has been reported in the past, but a lesion with a honeycomb‐like depression has never been reported before, making this an interesting lesion. We report here the mechanism of the depression and the morphology of the depression, together with a review of the literature.

## INTRODUCTION

Serrated lesions have been redefined in the World Health Organization (WHO) classification since 2019, and are divided into hyperplastic polyps, sessile serrated lesions (SSL), and traditional serrated adenomas.^1^ Furthermore, SSL is thought to be associated with dysplasia via a serrated pathway, resulting in SSL with dysplasia (SSLD), which may progress to colorectal cancer.^2^ On the other hand, there is no definition of serrated lesion with concavity in the WHO classification. Since Sobin reported inverted hyperplastic polyps in 1985, in which the epithelium of the crypts grows convex downward toward the submucosa,^3^ SSLs with similar morphology have been reported as inverted SSL.^4^

## CASE REPORT

**FIGURE 1:**
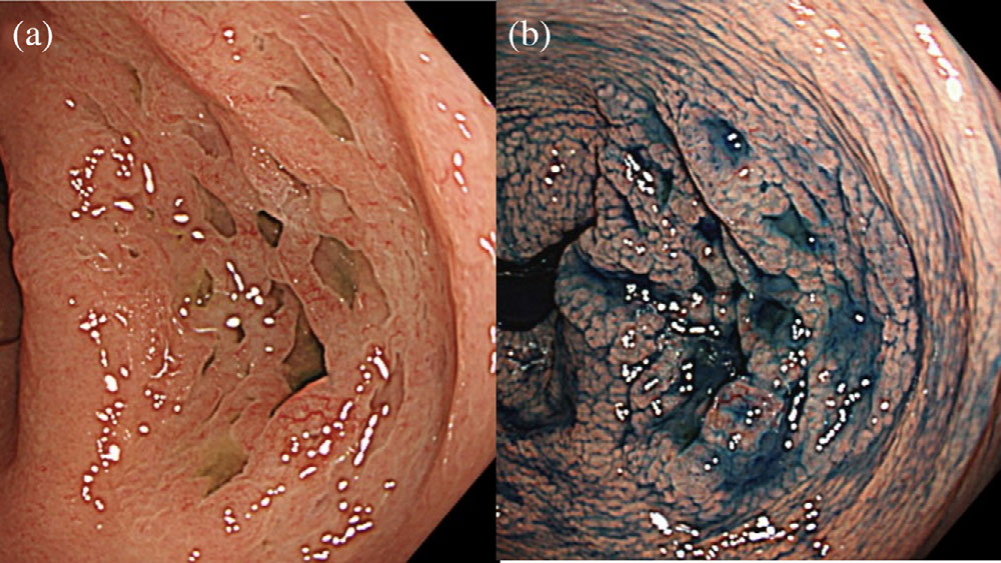
Close endoscopic examination of a honeycomb‐like inverted sessile serrated lesion. (a) White light image. Slightly faded lesions with multiple depressions of different sizes with indistinct borders are seen. (b) Chromoendoscopic image with indigo carmine. The lesions are well‐demarcated. The lesions appear somewhat soft with dehiscence.

**FIGURE 2:**
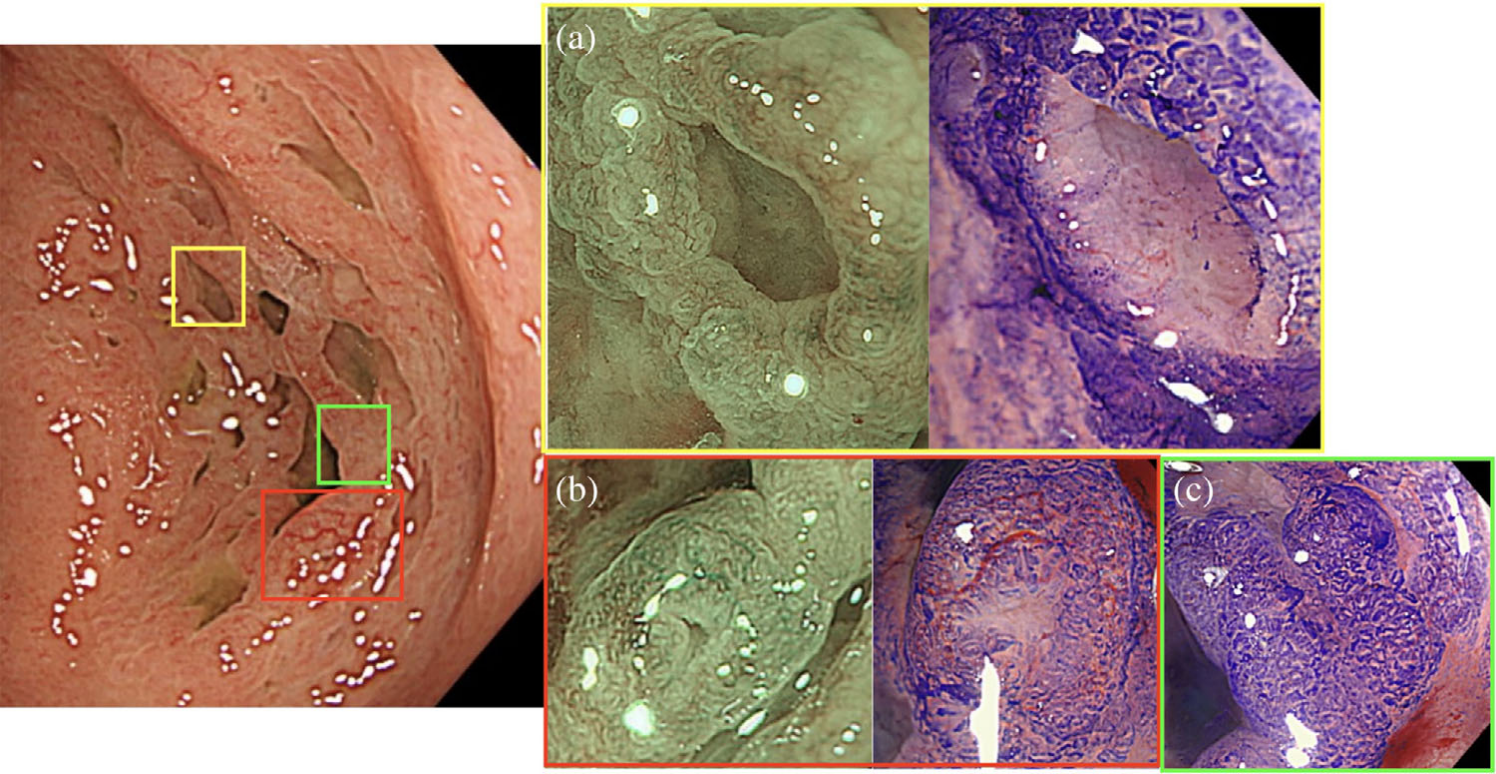
Endoscopic view of honeycomb‐like inverted sessile serrated lesion (SSL) with magnified narrow‐band imaging (NBI) and crystal violet staining. (a) The depressed area was observed and judged as Japan NBI Expert Team (JNET): type1; crystal violet staining was poor. (b) Partially dilated vessels were observed and judged as JNET: type2A. (c) The flat area had an open type II Pit pattern. (d) Border of the lesion. The border is clear.

The patient is a 63‐year‐old woman. No abnormal findings on physical examination or blood biochemical tests. She is receiving hormone therapy for breast cancer. No relatives have been treated for colorectal cancer and colorectal polyps. In 2022, an endoscopy of the lower gastrointestinal tract was performed by a local doctor for physical examination, and a 15‐mm‐sized SSL was found in the hepatic fold, and endoscopic submucosal dissection was performed at another hospital in March 2023. Serrated polyps were noted in two lesions, including the aforementioned and this case, and do not fit the 2019 WHO criteria for serrated polyposis syndrome diagnosis. Subsequently, a lower gastrointestinal endoscopy was performed in February 2024 for follow‐up, and a 35‐mm‐sized honeycomb‐like inverted SSL was observed in the ascending colon (Figure 1). The color was slightly faded in white light and some vasodilatation was observed. There were no surrounding diverticula, and multiple honeycomb‐like depressions were observed inside the lesion. The border between the lesion and the normal mucosa was indistinct under white light but became more distinct when indigocarmine was sprayed on the lesion, and a large amount of mucus adhesion was also observed. Narrow‐band imaging (NBI) magnification showed that the vessel pattern was generally invisible in the flat area, and the surface pattern was similar to the surrounding normal mucosa. On the other hand, the vessel pattern in Figure 2a showed dilated vessels with regular caliber and regular distribution. Based on the above, we diagnosed the patient as having mainly Japan NBI Expert Team (JNET) Type 1 and partly 2A. In Figure 2b, the depressed area, the vessel pattern was invisible and the surface pattern appeared to have a more dilated crypt, although it could not be observed in detail due to mucus adhesion. Crystal violet scattering showed that the pit pattern was open type II, which was suspicious for serrated lesions. The inside of the depression was difficult to evaluate due to the high mucus content and poor staining. The possibility of dysplasia could not be ruled out due to the presence of areas containing JNET 2A and insufficient observation of the interior of the depression by crystal violet staining, and the patient was referred to our hospital for endoscopic treatment because of the poor operability of the endoscope and the possibility of emergency surgery in the event of perforation during endoscopic treatment.

**FIGURE 3:**
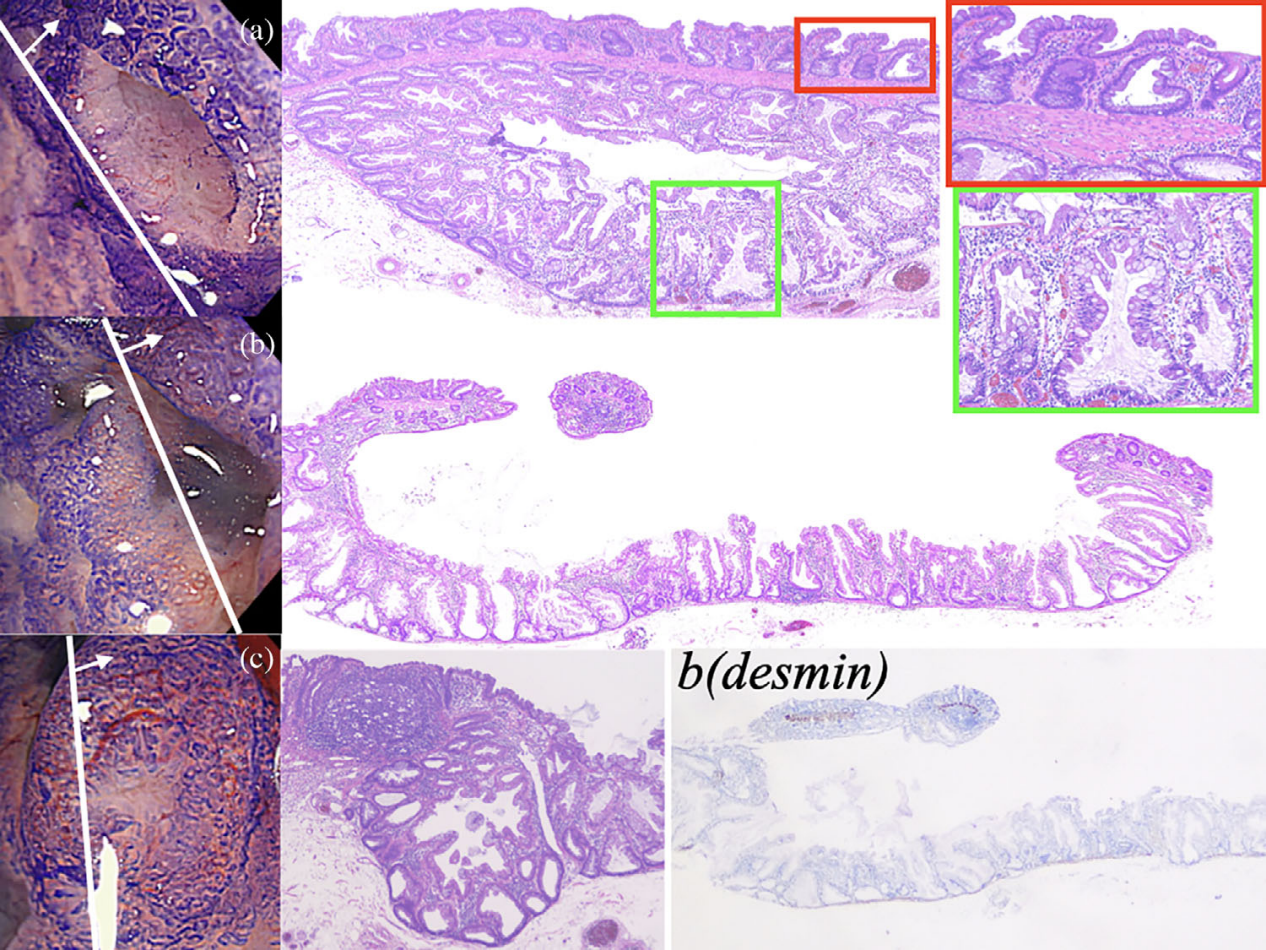
Endoscopic images contrasted with pathological images. (a) The area corresponding to Figure 2a. Serrated crypts are present in both the flat and depressed areas. The depression has more dilation of the crypt base, more goblet cells, and more mucus production than the flat area. (b) Large depressed area. The muscularis mucosae is thinned by downward pressure, and some destruction of the muscularis mucosae can be seen by desmin staining. (c) The area corresponding to Figure 2b. Lymphatic follicles are observed, but no dysplasia is seen.

**FIGURE 4 deo270106-fig-0004:**
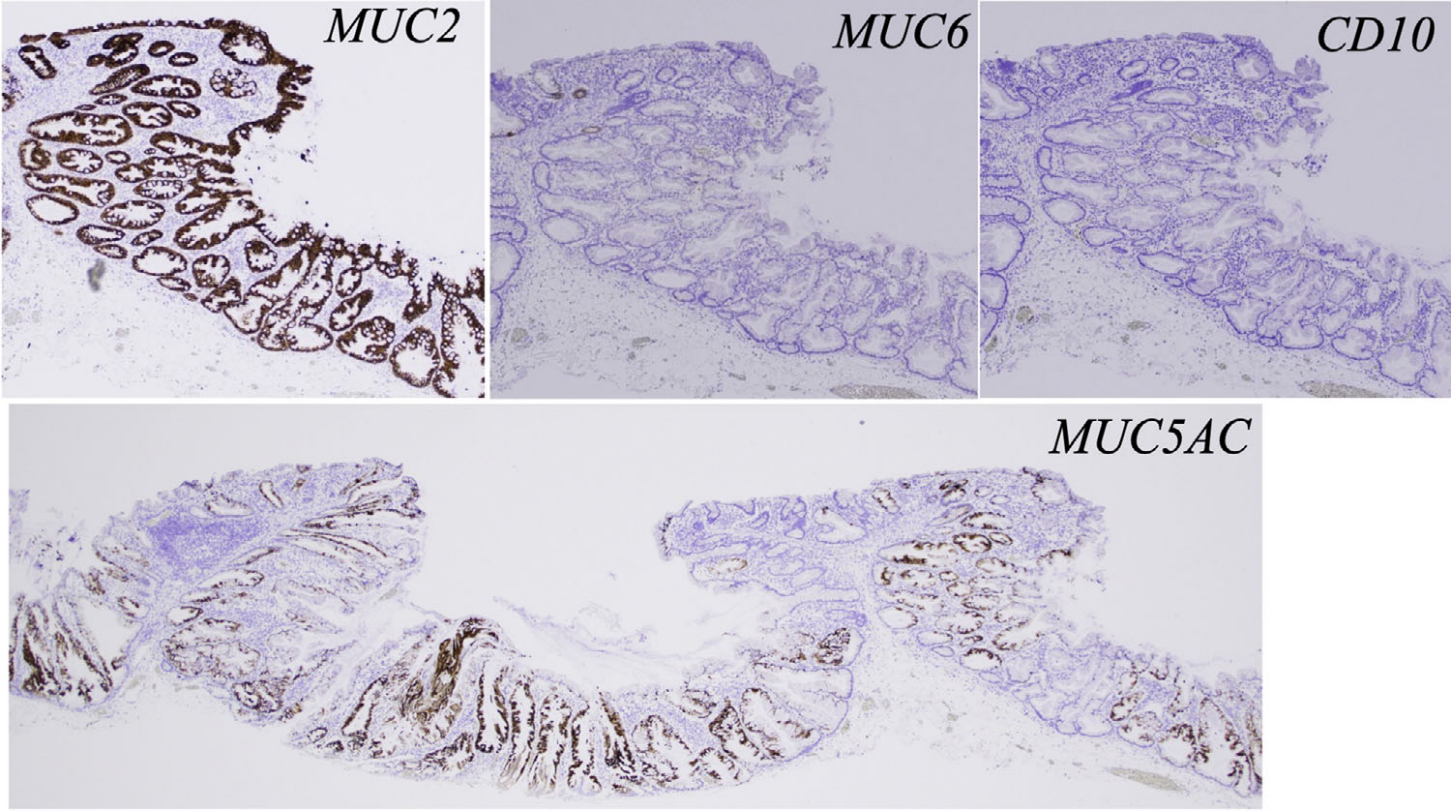
Immunostained pathological findings of the resected specimen. MUC2 was positive, MUC5AC was positive in some areas, and MUC6 and CD10 were negative. MUC5AC shows staining from the crypt opening to the middle of the depression, with less staining at the crypt base.

Colorectal endoscopic submucosal dissection was performed in April 2024 and completed without complications. The resected lesion was divided into 12 sections. Both flat areas and depressed areas show horizontal growth along the muscularis mucosae, serrations extending into the crypt base, and asymmetrical proliferation, which was the basis for the diagnosis of SSL in WHO Classification 2019. In addition, the area diagnosed as JNET Type 2A on NBI magnification prior to endoscopic treatment (Figure 3c) showed lymphatic follicles and no obvious dysplasia. The same was true in the other sections, and the diagnosis of SSL with inverted growth was made. On the other hand, the depressed area showed more pronounced dilation of the crypt base than the flat area. In addition, the crypts in the depressed area had more goblet cells and more mucus production, and the space between the crypts was narrower with the dilation of the crypt base. Desmin staining revealed that the muscularis mucosae layer was thin and partially destroyed (Figure 3b). Additional immunostaining for MUC2, MUC5AC, MUC6, and CD10 was performed (Figure 4). The results were positive for MUC2, partially positive for MUC5AC, and negative for MUC6 and CD10; MUC2 positivity indicates a colonic mucosal origin; MUC5AC is a marker found in the gastric mucosa, but has been reported to be positive in many cases of SSL.^5^ MUC5AC shows staining from the crypt opening to the middle of the depression, with less staining at the crypt base. This indicates that goblet cells are broken at the crypt base, but remain at the opening, indicating that the cytoplasm is preserved.[Fig deo270106-fig-0004]

## DISCUSSION

Inverted SSL is defined as the epithelium of the crypts growing convex downward toward the submucosa. Inverted SSL can be further divided into two pathological patterns: expansive growth type (EGT) and infiltrating growth type.^8^ EGT is a type of infiltration that pushes down the muscularis mucosae, while infiltrating growth type slips through the gaps between the muscularis mucosae and enters the submucosa and lymph follicles. The only inverted SSL that showed endoscopic depression was EGT.

The frequency of inverted SSL varied considerably, ranging from 1.1% in Lee et al.^6^ to 10.4% in Kawasaki et al.^7^ and 35.6% in Takashima et al.^8^ Takashima et al report was limited to EGT, the frequency was 12.5%. The frequency of EGT is considered to range from 1.1% to 12.5%, but the difference is still wide, future studies are needed to determine regional differences.

The morphology of the depression has rarely been reported, but most of them show a large depression in the center of the lesion,^4^, ^7^, ^8^, ^9^ and there have been no reports of multiple depressions of different sizes. Therefore, we defined “honeycomb‐like inverted SSL” as a form of inverted SSL in which four or more small or large depressions are observed on endoscopy at this hospital. It will be necessary to compile patient background data for inverted SSLs that fit this definition in the future.

As for the cause of SSL depression, it has been reported that crypt shape may be involved.^9^ The crypts in the depression of the inverted SSL remain largely the same at the opening, but the base is considerably dilated and frustoconical shape. The arrangement of the frustoconical crypts is thought to push the muscularis mucosae convexly downward. Furthermore, columnar crypts that do not have a wide base are not depressed when aligned, but are flat.

The reason why the crypt base is dilated may be related to the amount of goblet cells. Crypts in the depressed area have many goblet cells, and in some crypt bases, goblet cells are broken and released as mucus. The mucus is laterally compressed to form inverted T‐shaped and/or L‐shaped crypts and is also compressed downward pushing down the muscularis mucosae. Although mucus is draining upward, the multiple goblet cells near the crypt opening are relatively preserved and are not as dilated as at the base. In other words, we consider that the crypt base is dilated because more mucus is expelled from the base, but there is resistance in the upward direction.

It is necessary to distinguish it from a colonic diverticulum as a differential disease, it is important to observe the presence of mucus adhesion and the interior of the depression before treatment. Although there is no clear report on the carcinogenicity of inverted SSL, it is suggested that malignant transformation of inverted SSL may immediately lead to invasive carcinoma because the muscularis mucosae are pushed downward,^10^ and it is important to distinguish between SSLDs. Honeycomb‐like inverted SSLs such as the one in this case are likely to be large lesions, measuring approximately 30 mm, and may require treatment with endoscopic submucosal dissection.

This is the first report of a special morphology called “honeycomb‐like inverted SSL” in the world. The reason for this special morphology is unknown but thought to be related to the fact that the crypts in the depressed area were frustoconical shaped with many goblet cells, while the crypts in the flat area were conically shaped with few goblet cells. Since this is a single case study, it is necessary to compare the amount of goblet cells and crypt shape between flat and concave areas if other honeycomb‐like inverted SSLs are found.

## CONFLICT OF INTEREST STATEMENT

None.
